# Bridging the gap in heart failure management: the effect of a cross-disciplinary intervention on guidelines-directed medical therapy in primary care

**DOI:** 10.1093/eschf/xvag036

**Published:** 2026-01-20

**Authors:** Veronica Milos Nymberg, Anton Grundberg, J Gustav Smith, Kristina Sundquist, Oscar Braun

**Affiliations:** Center for Primary Health Care Research, Department of Clinical Sciences Malmö, Lund University, Building 28, Floor 11, Jan Waldenströms Street 35, Malmö 205 02, Sweden; University Clinic Primary Care, Skåne University Hospital, Region Skåne, Sweden; Center for Primary Health Care Research, Department of Clinical Sciences Malmö, Lund University, Building 28, Floor 11, Jan Waldenströms Street 35, Malmö 205 02, Sweden; Department of Cardiology, Clinical Sciences, Lund University and Skåne University Hospital, Lund, Sweden; Department of Molecular and Clinical Medicine, Institute of Medicine, University of Gothenburg, Gothenburg, Sweden; Wallenberg Center for Molecular Medicine and Lund University Diabetes Center, Lund University, Lund, Sweden; Center for Primary Health Care Research, Department of Clinical Sciences Malmö, Lund University, Building 28, Floor 11, Jan Waldenströms Street 35, Malmö 205 02, Sweden; University Clinic Primary Care, Skåne University Hospital, Region Skåne, Sweden; Department of Cardiology, Clinical Sciences, Lund University and Skåne University Hospital, Lund, Sweden

**Keywords:** GDMT, Adherence, Primary care, Heart failure, Cross-disciplinary programme

## Abstract

**Background and aims:**

Guideline-directed medical therapy (GDMT) for heart failure (HF) is underutilized in primary care, particularly among older adults with chronic stable HF. This prospective quality improvement study, Heart Failure in Southern Sweden (HISS), evaluated the impact of a cross-disciplinary implementation project combining cardiology and primary care expertise to enhance GDMT adherence and reduce healthcare contacts.

**Methods:**

Twenty primary health care centres in southern Sweden participated, recruiting 587 patients diagnosed with HF (mean age 79 years) between 2021 and 2023. The intervention involved case-based educational conferences with cardiologists and general practitioners, individualized treatment recommendations, and follow-up monitoring. Medication use and healthcare contacts were assessed 6 months before and after the intervention.

**Results:**

GDMT use (defined as quadruple therapy according to the 2022 guidelines) increased from 20.8% at baseline to 37.7% post-intervention (*P* < .001) among patients with HF with reduced ejection fraction (HFrEF), and from 12.4% to 17.8% (*P* = .020) among patients with mildly reduced ejection fraction (HFmrEF). The uptake of sodium–glucose co-transporter-2 inhibitors (SGLT2i) improved significantly across all HF types, while angiotensin receptor-neprilysin inhibitors (ARNI) increased among HFrEF patients. Beta-blocker use declined in patients with HF with preserved ejection fraction. The total number of ambulatory healthcare contacts decreased following the intervention, while the hospitalizations remained unchanged.

**Conclusions:**

The HISS study demonstrates that a cross-disciplinary, case-based educational intervention was associated with improved GDMT adherence (especially SGLT2i and ARNI) and reduced ambulatory healthcare utilization in primary care patients with chronic stable HF. These findings underscore the importance of bridging the gap between specialist and primary care to optimize HF management.

## Graphical Abstract

**Graphical Abstract:**
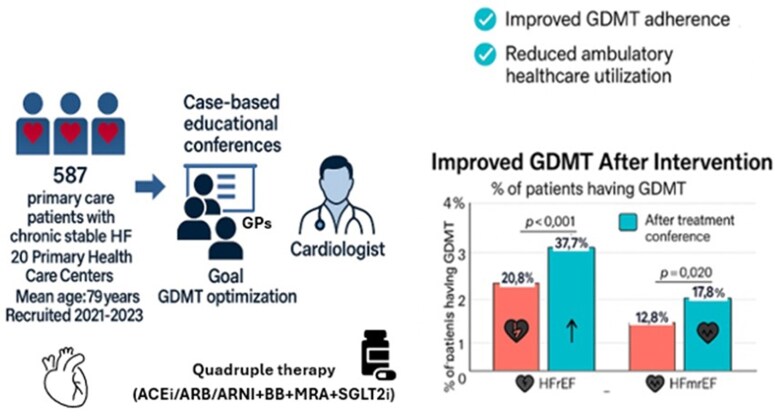
The implementation of the dialogue between primary care and cardiology was associated with improved GDMT adherence, especially for SGLT2i, and a decreased number of ambulatory health care contacts.

## Introduction

Following recent advances in the treatment of heart failure (HF), international guidelines advocate prompt initiation of foundational evidence-based therapies to improve mortality, morbidity and quality of life,^1–3^ referred to as guideline-directed medical therapy (GDMT). Even though the guidelines are clear and early benefits of GDMT have been proven,^4^ the implementation of the recommendations in primary health care settings has been challenging.^5^

Epidemiological data show that the global burden of HF is increasing, particularly for patients with HF with preserved ejection fraction (HFpEF), due to the aging population and improved treatment and survival of patients with ischaemic heart disease.^6^ Most of these patients are treated in primary care settings^7^ and are older.^8^ Therefore, it has been argued that the implementation of guidelines for the management of HF needs to be addressed from the primary care perspective.^7^

Suboptimal implementation of GDMT in primary care can be explained by several factors. Unlike other chronic life-threatening diseases, such as cancer, HF often presents with stable symptoms in primary care, meaning that the risk of morbidity and mortality is often underappreciated by the clinicians.^9^ The previously suggested approach with sequential initiation and titration of different drug classes is mostly time-consuming. Additionally, studies have shown that, unlike cardiologists, general practitioners (GPs) do not always feel comfortable with changes in HF medication.^10^ It is therefore not surprising that the updated GDMT strategy,^11^ advocating rapid treatment initiation and titration, can be challenging for GPs.

In Sweden, most patients with chronic HF are managed in primary care, where resources and infrastructure vary considerably.^12^ While nurse-led HF patient receptions seem to provide high-quality person-centred care and reduce hospitalizations, they are uncommon outside specialized care, and the availability of HF nurses in primary health care centres is limited and inconsistent.^12^ As GPs are trained to use a patient-centred approach, the updated guidelines, emphasizing a new personalized approach with patient profiling in the decision-making process^13^ could represent a paradigm shift in the management of HF patients. To bridge the gap between cardiology and primary care, and to improve adherence to GDMT in HF, interventions targeting GPs have been recommended.^5^

The general aim of the prospective implementation study Heart Failure in Southern Sweden (HISS) is to describe the effect of a multi-disciplinary quality improvement intervention with cardiological and primary care expertise on changes in GDMT and health care contacts in a cohort of primary care patients with HF.

## Methods

### Study design

The HISS project was a prospective implementation study within routine clinical practice in primary care with the goal to evaluate the impact of a case-based, cardiologist-supervised educational programme to improve GDMT adherence in a real-world setting.

### Setting and participants

Primary health care centres (PHCCs) in southern Sweden, both public and private, were invited to participate in the study by information meetings with the primary care managers. The PHCCs that were interested received a visit from a researcher and a biomedical analyst, with information about the study. Out of ∼160 PHCCs invited, the first 20 PHCCs who agreed to participate in the study were consecutively included between 2020 and 2023.

PHCCs recruited community-dwelling adult patients (18 years or older), based on International Classification of Disease (ICD-10) diagnosis codes I50, I11.0, I42 (excluding I42.1, I42.2) and I43. Patients living in nursing homes were excluded.

The investigation conforms with the principles outlined in the *Declaration of Helsinki.* Written informed consent was obtained from all participants. The project was approved by the Swedish Ethical Review Authority (Dnr 2019-03944).

### Recruitment

Each PHCC designated a research assistant (nurse or resident doctor) responsible for the patient recruitment and data collection. The PHCC manager was instructed to plan a case-based educational treatment conference with a cardiologist, gathering all GPs, resident doctors and HF nurse (if available) at the PHCC.

The research assistant identified eligible patients and sent letters of information with informed consent forms. Patients were invited to the primary care unit for blood sampling, blood pressure measurement, and electrocardiography (ECG), and were asked to sign the informed consent form if they wished to participate in the study. The research assistant collected data, including information about comorbidities, medications, blood results and other measurements in a Case Report Form (CRF), which had two parts: nurse-CRF and physician-CRF. The study variables were noted by the research assistant on the nurse-CRF part. If the research assistant was a HF nurse, she also registered the patient in the Swedish Heart Failure Registry (SwedeHF). Based on the available information in the nurse-CRF, the research assistant could make suggestions if the patient needed to be brought up at the treatment multi-disciplinary conference. The nurse-CRF was provided to the GP responsible for the patient. This allowed the treating physician at the PHCC to look more closely at the patient record before the treatment conference and prepare questions.

### The intervention

The participating PHCC convened a half-day for a mutually learning educational treatment conference where all the physicians from the PHCCs (GPs and resident doctors) and any HF nurse were invited to participate together with a cardiologist (O.B.) involved in the study. For the chosen patient cases, the nurse-CRF was reviewed together and recommendations for optimized treatment were discussed, based on the patient’s individual characteristics and needs. On the physician-CRF, the named GP attending the conference made and documented the final decision, i.e. indicating if the patient would receive optimized treatment. Once the physician-CRF was completed, all CRFs were collected by the research assistant, and the study folder was sent to our department at Lund University, Center for Primary Health Care Research, where a research nurse registered the data into the REDCap data management programme.

### Survey procedure, collection, and nature of data

The following data from the CRF forms were collected in the REDCap electronic database: sex, age, ICD-10, diagnosis codes ICD-10 (comorbidity), information on the completed echocardiography, results in terms of EF, disease classification (New York Heart Association-NYHA class), test results, blood pressure, heart rate, QRS length on most recent ECG, treatment, any change in treatment after the completed treatment conference, and reason for not changing the treatment (if applicable). EF-based subtypes were defined as follows: HF with preserved EF (HFpEF; EF ≥ 50%), HF with mildly reduced EF (HFmrEF; EF 41%–49%), and HF with reduced EF (HFrEF; EF ≤ 40%).^3^

The blood samples taken and sent to clinical chemistry were Hb, fP-glucose, cholesterol, triglycerides, LDL, HDL, ALAT, GT, bilirubin, iron, transferrin saturation, TIBC, ferritin, Na, K, creatinine, eGFR, and NT-proBNP.

For comparison analysis, data on medications (collected prescriptions from the pharmacy) and healthcare contacts 6 months prior to the inclusion and 6 months after the treatment conference were collected from the regional health care database.

Information about the following medication classes was collected: Angiotensin-Converting Enzyme Inhibitors (ACE-i) ATC-code C09A (including drug combinations C09B), Angiotensin II Receptor Blockers (ARB) ATC-code C09C (including combinations C09D), Angiotensin Receptor-Neprilysin Inhibitors (ARNI) ATC code C09DX, Beta-Adrenergic Blockers (BB) ATC code C07A, Mineralocorticoid Receptor Antagonists (MRA) ATC code C03DA, Loop diuretics ATC code C03CA, Sodium–glucose co-transporter-2 (SGLT2-i) ATC code A10BK. The combination of at least one of ACE-i, ARB and ARNI was defined as RASi.

Healthcare contacts registered with relevant diagnoses within 6 months before and within 6 months after the treatment conference were defined as the following:

1. Ambulatory care: physical visits or telephone contacts with a physician or a nurse in primary care, specialized care (open patients department at a hospital clinic);
2. Hospital care: emergency visits at a hospital and hospitalizations.

Relevant contacts were those documented in the electronic medical record with at least one of the following ICD-10 diagnosis codes: I (all cardiovascular diseases), R06 (dyspnoea), R60 (oedema), R05 (cough), R07 (chest pain), R429 (dizziness and vertigo), R00 (palpitations), R55 (fainting), and E87 (electrolyte disorders).

### Data analysis

#### Power calculations

Sample size calculations were based on detecting clinically significant effects using the cardiology-supervised educational programme defined as an increase of 10% in the number of patients treated with a combination of RASi and beta-blockers at a follow-up after 6 months. Based on the assumption that approximately half of the patients with a correct HF diagnosis provided consent to participate in the study (∼50 patients per health care centre) and with a statistical power of 80% (β = 0.2), a significance level of α = 0.05, and an intraclass correlation coefficient (ICC) of 0.1, at least 17 health care centres (850 patients) were needed to be included.

#### Data analysis

McNemar’s χ^2^ tests were used to assess the change in prescription of medication classes before study inclusion and after treatment conference, depending on HF type.

Changes in healthcare contacts from before and after treatment conference, were analysed using negative binomial mixed model for repeated measures and presented with estimates of incidence rate ratios (IRR) with 95% confidence intervals (CI), for any kind of visit and depending on ambulatory/hospital care and to physician or nurse. All statistical analyses were performed with R version 4.4.2 (R Core Team, 2024).

## Results

In total, 587 adult patients diagnosed with HF from 20 PHCC in different sociodemographic areas were recruited. Four recruiting PHCCs had a HF nurse, with 93 patients (15, 8%) in total included in the study and registered to SwedeHF. The median age was 79 years (range 35–96), with a majority (46%) being >80 years old.

A total of 558 (95%) of the included participants had available data on left ventricular ejection fraction, with most of them (two thirds) having undergone an echocardiography within 3 years prior to the inclusion in the study (median 2 years). HFpEF was most prevalent (42%), followed by HFmrEF (30%) and HFrEF (28%) with no difference in distribution between men and women. Only 15% of the cohort was registered in the Swedish Heart Failure register (SwedeHF) at recruitment.

**Figure 1:**
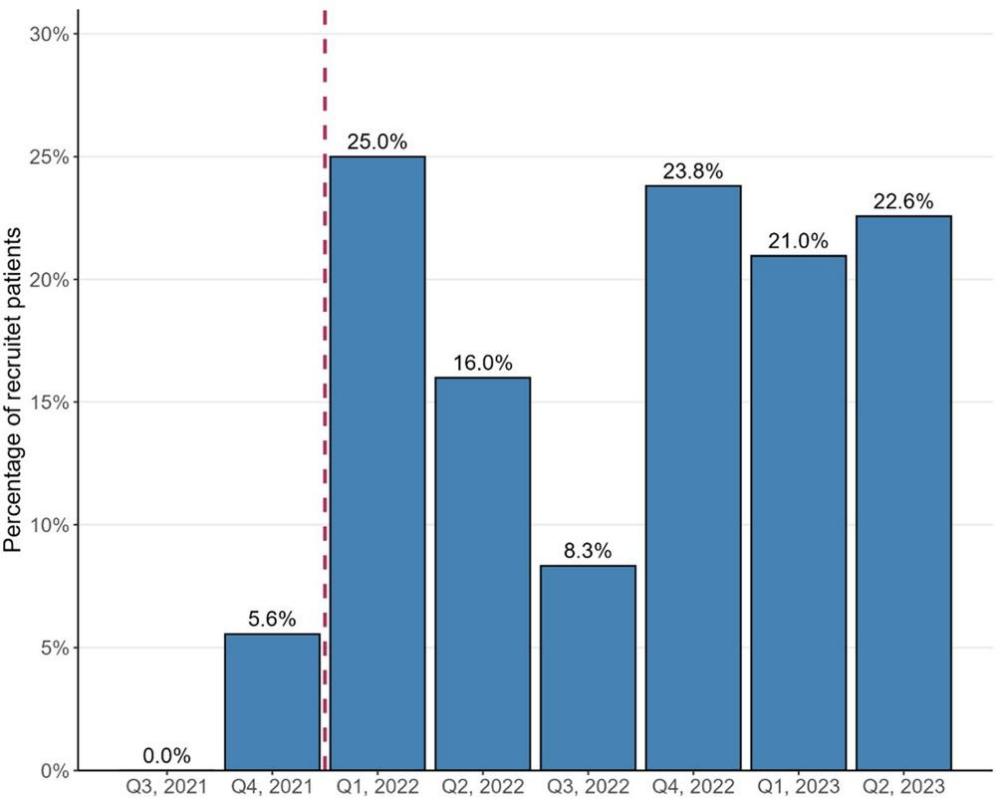
Timeline for percentage of recruited patients receiving GDMT at baseline during the recruitment from 2021 to 2023 in each quarter of a year (=Q). New GDMT became available after the first quarter of 2022 (marked by the dotted line)

Most of the patients (81%) were included between 2022 and 2023 and 19% between 2020 and 2021 (the start of the COVID-19 pandemic), with no statistically significant difference observed between those included before and after 2022 when comparing the distribution of different EF subtypes or GDMT. No clear trend of increasing adherence to the new recommended GDMT at baseline was seen during the recruitment period between the end of 2021 and the end of 2023 (*Figure 1*).

**Figure 2:**
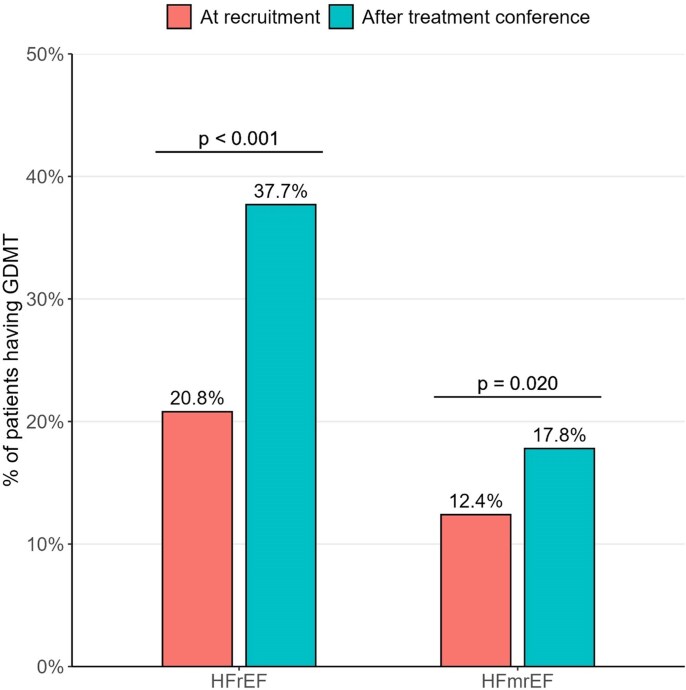
Percentage of patients with HFrEF and HFmrEF having GDMT according to the new ESC guidelines (2022) with quadruple therapy including a combination of RASi, BB, MRA, and SGLT2i. Comparison between baseline and after the treatment conference, tested using McNemar’s χ^2^ tests

Group comparisons between baseline and follow-up showed that the percentage of patients receiving GDMT (defined as quadruple therapy) increased significantly from 20.8% to 37.7% in the HFrEF group (*P* < .001), and from 12.4% to 17.8% in the HFmrEF group (*P* = .020) (see *Figure 2* and Supplementary Table S1).

**Figure 3:**
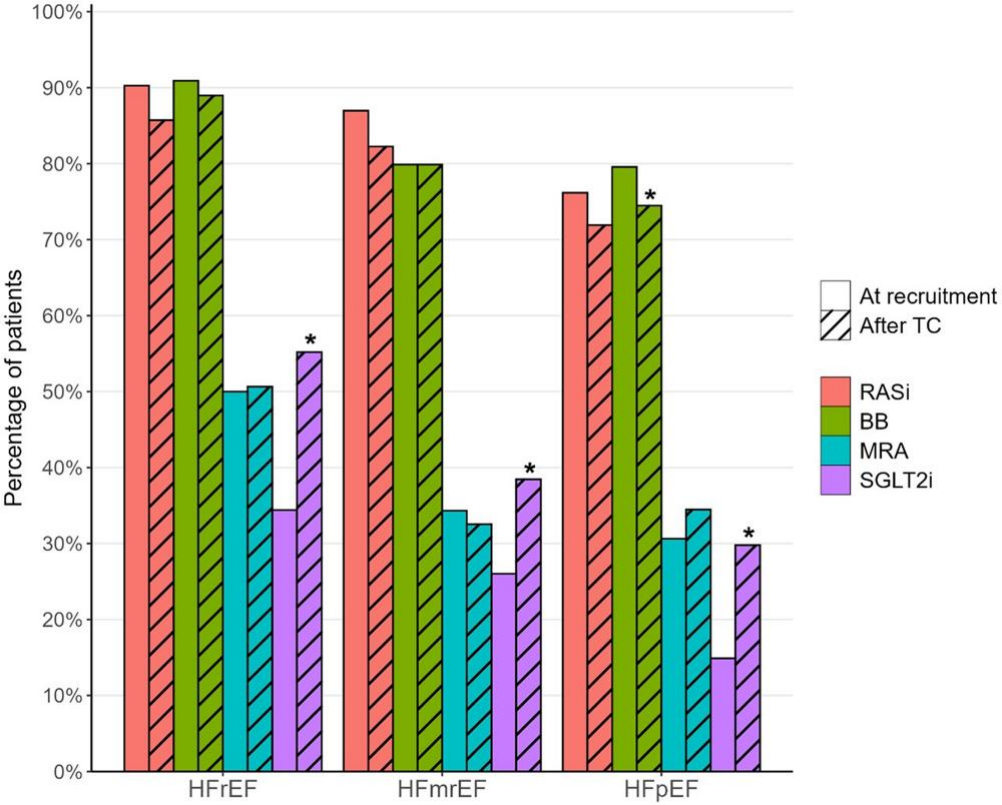
The distribution of the number of the foundational GDMT drug classes before and after the intervention in each EF group. Asterisks indicate significant change

The distribution of the medication classes in different EF groups at baseline and at follow-up is shown in *Table 1* and *Figure 3*. Group comparisons before/after the treatment conference showed a substantial and significant increase in the percentage of patients receiving SGLT2i across all EF categories. In the HFrEF group, a decrease in the percentage of patients receiving ACEi was observed along with nominal trends towards decreased ARB use and increased ARNI use. In the HFpEF group, the percentage of patients receiving BB declined significantly.

**Table 1:** Number of patients with different medication classes at recruitment and after treatment conference per HF type

Heart failure		ACEi	ARB	ARNI	RASi	BB	MRA	SGLT2i	Loop diuretics
HFrEF, *n* = 154	Before	74 (48.1)	58 (37.7)	16 (10.4)	139 (90.3)	140 (90.9)	77 (50.0)	53 (34.4)	84 (54.5)
	After	62 (40.3)	51 (33.1)	21 (13.6)	132 (85.7)	137 (89.0)	78 (50.6)	85 (55.2)	75 (48.7)
	Change in proportions	−7.8 (−13.6, −3.1)	−4.5 (−10.4, 0.8)	3.2 (−0.2, 7.7)	−4.5 (−10.4, 0.8)	−1.9 (−6.9, 2.6)	0.6 (−6.2, 7.6)	20.8 (13.1, 28.8)	−5.8 (−13.2, 1.3)
	*P*-value	.003	.090	.059	.090	.366	.847	<.001	.106
HFmrEF, *n* = 169	Before	88 (52.1)	56 (33.1)	4 (2.4)	147 (87.0)	135 (79.9)	58 (34.3)	44 (26.0)	80 (47.3)
	After	82 (48.5)	53 (31.4)	4 (2.4)	139 (82.2)	135 (79.9)	55 (32.5)	65 (38.5)	71 (42.0)
	Change in proportions	−3.6 (−8.8, 1.2)	−1.8 (−6.0, 2.0)	0.0 (−2.2, 2.2)	−4.7 (−10.2, 0.2)	0.0 (−5.5, 5.5)	−1.8 (−7.5, 3.8)	12.4 (6.7, 18.9)	−5.3 (−11.9, 1.0)
	*P*-value	.134	.317	.999	.059	.999	.513	<.001	.137
HFpEF, *n* = 235	Before	95 (40.4)	86 (36.6)	2 (0.9)	179 (76.2)	187 (79.6)	72 (30.6)	35 (14.9)	115 (48.9)
	After	88 (37.4)	82 (34.9)	1 (0.4)	169 (71.9)	175 (74.5)	81 (34.5)	70 (29.8)	108 (46.0)
	Change in proportions	−3.0 (−7.2, 0.9)	−1.7 (−5.1, 1.4)	−0.4 (−2.4, 1.2)	−4.3 (−9.0, 0.2)	−5.1 (−9.9, −0.7)	3.8 (−0.7, 8.6)	14.9 (9.5, 20.7)	−3.0 (−9.0, 2.9)
	*P*-value	.127	.248	.317	.059	.023	.095	<.001	.317
HF with EF	Before	9 (31.0)	10 (34.5)	1 (3.4)	20 (69.0)	22 (75.9)	8 (27.6)	0 (0.0)	11 (37.9)
unknown, *n* = 29	After	9 (31.0)	9 (31.0)	1 (3.4)	19 (65.5)	22 (75.9)	9 (31.0)	6 (20.7)	10 (34.5)
	Change in proportions	0.0 (−11.7, 11.7)	−3.4 (−17.2, 8.7)	0.0 (−11.7, 11.7)	−3.4 (−17.2, 8.7)	0.0 (−14.4, 14.4)	3.4 (−8.7, 17.2)	20.7 (6.6, 38.4)	−3.4 (−21.3, 14.1)
	*P*-value	.999	.317	.999	.317	.999	.317	.014	.655

Data are presented as *n* (%) and changes in proportions as %. Differences tested using McNemar’s χ^2^ tests.

The most common reasons for not changing the medication after the treatment conference, as documented in free text by the GPs in the CRF, were medication side-effects (as bradycardia or low blood pressure), low patient compliance, or lack of severe symptoms (NYHA I).

The comparison between healthcare contacts before and after the treatment conference showed a decrease in the number of total ambulatory contacts per month at both physicians and nurses in the period after the treatment conference, but no difference in hospitalizations (*Table 2*).

**Table 2:** Healthcare visits for relevant diagnoses^a^ per 6-month period, before and after treatment conference

	All contacts				Ambulatory contacts				Hospitalizations/contacts with emergency care			
All patients *N* = 587	Before	After	IRR (95% CI)	*P*-value	Before	After	IRR (95% CI)	*P*-value	Before	After	IRR (95% CI)	*P*-value
All contacts			0.77 (0.70, 0.84)	<.001			0.76 (0.69, 0.83)	<.001			1.13 (0.85, 1.50)	.400
Min—max	0–42	0–83			0–38	0–83			0–5	0–4		
Mean (SD)	3.95 (4.21)	3.28 (5.32)			3.80 (3.96)	3.10 (5.10)			0.16 (0.54)	0.18 (0.60)		
Median (IQR)	3.00 (4.00)	2.00 (3.00)			3.00 (4.00)	2.00 (3.00)			0.00 (0.00)	0.00 (0.00)		
Physician contacts			0.78 (0.70, 0.85)	<.001			0.76 (0.67, 0.85)	<.001			1.13 (0.85, 1.50)	.400
Min—max	0–19	0–20			0–16	0–16			0–5	0–4		
Mean (SD)	2.57 (2.59)	2.04 (2.53)			2.41 (2.33)	1.87 (2.20)			0.16 (0.54)	0.18 (0.60)		
Median (IQR)	2.00 (3.00)	1.00 (3.00)			2.00 (2.00)	1.00 (3.00)			0.00 (0.00)	0.00 (0.00)		
Nurse contacts			0.76 (0.66, 0.88)	<.001			0.76 (0.66, 0.88)	<.001				
Min—max	0–29	0–75			0–29	0–75				N/A		
Mean (SD)	1.39 (2.67)	1.23 (4.06)			1.39 (2.67)	1.23 (4.06)						
Median (IQR)	1.00 (2.00)	0.00 (1.00)			1.00 (2.00)	0.00 (1.00)						

IRRs with 95% CIs and *P*-values estimated using negative binomial mixed model for repeated measures.

^a^ICD-10 diagnosis codes: I (all cardiovascular diseases), R06 (dyspnoea), R60 (oedema), R05 (cough), R07 (chest pain), R429 (dizziness and vertigo), R00 (palpitations), R55 (fainting), and E87 (electrolyte disorders).

## Discussion

This cross-disciplinary quality improvement study demonstrated a significant increase in the use of GDMT in patients with HFrEF and HFmrEF after the cross-disciplinary treatment conference. Regarding specific medication classes, we found an increase in SGLT2i for all HF types, a decrease in the use of BB for patients with HFpEF, and a decrease of the use of ACEi with a trend towards increased use of ARNI in patients with HFrEF. Despite a lengthy recruitment period, which overlapped with the introduction of updated Swedish guidelines in the beginning of 2022, the use of GDMT at recruitment remained constant over time, indicating that the increase after the treatment conference might be attributable to increased attention to some patients after the cross-disciplinary discussion with the cardiologist. The number of healthcare contacts decreased after the treatment conference but was mostly related to a decrease in ambulatory contacts. The findings indicate that the increase in ARNi might have been related to prescriptions during the hospital contacts, which is in line with current Swedish GDMT for HF. The decrease in ACEi and ARB among HFrEF patients may partly be attributable to the therapeutic switch to ARNi. Unfortunately, patient-level transition data (e.g. ACEi/ARB to ARNI) were not available, as medication information was derived from collected prescription data in the regional healthcare database without sequencing details. Therefore, we cannot confirm whether this decline reflects therapeutic switching or discontinuation without replacement. Furthermore, the decrease in the use of ACEi and ARB could also partly be due to clinical decisions based on patient characteristics such as frailty, renal function, or blood pressure. However, sub-group comparison analyses between the groups of HFrEF patients with and without ACEi or ARB after the treatment conference showed no significant differences in frailty indicators such as age, systolic blood pressure or kidney function (that might otherwise have explained medication withdrawal or GP’s decision to not initiate treatment with these drug classes).

Since 2021, robust evidence for the treatment of HFmrEF and HFpEF with SGLT2i has been obtained. The uptake of SGLTi treatment for HFrEF has been more pronounced than treatment with other drug classes^14^ and hopefully this will translate into a faster uptake for patients with EF > 40% as well. However, these quite recent findings have probably not been fully disseminated to primary care. Most real-world evidence regarding HF treatment stems from large registries where data from primary care settings is highly under-represented, which is evident from our study where only 15.6% of the patients were registered in SwedeHF. Thus, interventions to increase and optimize treatment in primary care settings are needed.

### Strengths and limitations

This is the first study describing the adherence to GDMT in Swedish primary care, and changes over time after a multi-disciplinary quality improvement intervention including both cardiology and primary care expertise. To the authors’ knowledge there are no other intervention studies in primary care with a focus on health care contacts as an outcome, which is an additional strength of the study.

However, the study lacks a control group, which is a major limitation. Implementing a stepped-wedge or cluster-randomized design would have strengthened the study but would have required substantial infrastructure, extended timelines, and additional funding beyond what was available for this pragmatic initiative. Because the intervention was integrated into existing workflows and designed to be deployed rapidly using available personnel and systems, adopting a more complex randomized design was not practical. Given these constraints, we selected an implementation-focused approach that aligned with the real-world setting and the project’s quality improvement goals. Also, while the intervention did not involve withholding GDMT, delaying the educational component for certain clusters could have perpetuated suboptimal care during the study period. Given the high-risk population and the urgency to improve adherence to guideline-directed therapy, we prioritized rapid implementation. Although patients were nested within PHCCs, cluster-adjusted analyses were not performed because the study used a pre–post design focused on within-patient changes; the ICC assumption (0.1) was applied only for initial power calculations. This study contains multiple tests and, therefore, *P*-values should be interpreted in the context of effect sizes and their clinical relevance.

The extended recruitment period, which was partially attributable to the COVID-19 pandemic in 2021–2022, resulted in the inclusion of 20 PHCCs and 587 patients, which was less than originally planned. Although the final patient count was below the initial target determined by the power calculation, the observed increase of over 10% in GDMT adherence indicates that the study maintained adequate statistical power. A limitation of this study is its single-arm, pre–post design without a control group, which restricts causal inference. The study period overlapped with the introduction of updated ESC guidelines and the adoption of quadruple therapy in 2021–2022, as well as ongoing dissemination efforts through local educational meetings and published materials. These secular trends and concurrent initiatives may have contributed to improvements in GDMT independent of our intervention. To explore this possibility, we examined baseline data across the recruitment period and found no clear upward trend in GDMT adherence prior to the intervention, suggesting that the observed increase post-conference was not solely driven by guideline dissemination. Nevertheless, the influence of evolving evidence and broader implementation strategies cannot be excluded and should be considered when interpreting the results. The model of the intervention, including both cardiology expertise but also the GP responsible for the patient at the PHCC, offered the possibility to make the treatment decision in a patient-centred manner, with good knowledge of the patients’ individual characteristics and needs. While the cardiologist had knowledge about GDMT and latest evidence in the field of HF, the GP could make the final decision not only based on the relatively fewer cases discussed during the treatment conference, but also for other patients included in the study. This model might have bridged the gap between specialized care and primary care, empowering the GPs in their decisions to initiate optimized treatment for HF.

The variability in resources across primary care, including the limited and inconsistent availability of HF nurses among participating PHCCs, may have influenced baseline quality of care and the intervention’s effect. This heterogeneity should be considered when interpreting the findings and assessing generalizability to other primary healthcare settings. Selection bias is a potential limitation, as the first 20 PHCCs that volunteered among ∼160 invited centres were included. These centres may have had higher motivation or better infrastructure for HF care compared with the non-participating PHCCs. Additionally, nursing home residents were excluded, which limits generalizability to frailer populations with more complex care needs. These factors should be considered when interpreting the findings and applying them to other settings.

A limitation of this study is that GDMT adherence was assessed at the class level based on collected prescriptions, without evaluating titration to guideline-recommended target doses or persisting treatment over time. This approach reflects the pragmatic design and data source constraints; medication data were retrieved from the regional healthcare database, which does not capture dosing or titration steps. Collecting such granular information would have required repeated follow-up visits and infrastructure beyond the scope and capacity of this quality improvement initiative. Our primary aim was to improve initiation of foundational GDMT classes in the most often older, community-dwelling patients who are mainly treated in primary health care, a critical first step towards better outcomes in a population where underuse remains a major barrier. While dose optimization is clinically important, as highlighted by STRONG-HF,^4^ future studies should incorporate metrics such as defined daily dose or days-covered to provide a more comprehensive assessment of treatment optimization and adherence.

Healthcare contacts were included as a secondary outcome to explore whether improved GDMT adherence could reduce the symptom burden and thereby decrease healthcare utilization. However, the 6-month observation window was chosen pragmatically and may have been too short to capture the effect of changes in medication. Moreover, reductions in ambulatory contacts could reflect factors unrelated to the intervention, such as staffing or coding changes, scheduling practices, or regression to the mean. Reverse causality is also possible, as patients with greater care needs may have received more intensive medication optimization.

A potential limitation of this study is the Hawthorne effect, as the presence of a cardiologist during the educational treatment conferences may have influenced the behaviour of participating clinicians beyond the intended intervention. This effect could have led to greater attention to HF management simply because clinicians were aware of being observed and supported by a specialist. Furthermore, the cardiologist facilitating the intervention was also a co-author (O.B.), which may have introduced additional bias through enhanced engagement or motivation among participating clinics. While this involvement ensured clinical accuracy and relevance of recommendations, it should be acknowledged as a factor that may limit generalizability to settings without direct specialist participation.

Over the past years, a substantial body of evidence has established the use of the four foundational pharmacological treatments for HFrEF, each shown to reduce mortality and morbidity while improving quality of life.^11^ Despite clear guideline recommendations, the implementation of these therapies has been slow. However, recent data from Sweden indicate an encouraging increase in the use of GDMT, which is also reflected in improved patient outcomes.^15,16^ However, older patients are probably overrepresented in primary care but often undertreated and, despite improvements in medical treatment for HF, the overall prognosis for patients with HFrEF remains poor, underscoring the urgent need to further enhance the uptake and optimal use of these proven, life-saving treatments.^15,16^

Our results showed high use of ACEi and BB in patients with reduced EF, in line with previous register-based data.^17,18^ However, temporal trends show that improvements and survival in the last generation might level off, suggesting that efforts to increase GDMT implementation should increase.^17^

We found no other intervention studies in primary care populations with HF, focusing on health care contacts as outcomes. However, several studies have investigated interventions aimed at improving medication adherence and optimizing therapy in primary care patients with HF. A recent systematic review found that interdisciplinary HF clinics, often led by nurses and pharmacists, were the most consistently effective interventions for optimizing GDMT, particularly for up-titration of beta-blockers and RASi. Other strategies, such as electronic health record alerts, audits, and education-based initiatives, showed some benefit but produced variable and less consistent results across studies.^19^ The study showed that a multi-disciplinary approach has effect on optimizing GDMT but included only patients with HFrEF managed by specialized HF clinics (inpatient or outpatient clinics), who are often more ill and prone to hospital admissions compared with most patients with HF in primary care. The patients in our study had chronic stable HF (predominantly HFpEF), representative for most HF patients handled in primary care. We believe that our pragmatic approach, well adapted to the primary care context, might be a suitable care model for patients with stable chronic HF in primary care.

Other studies have included Multi-disciplinary Management Programs,^20^ Physician-Targeted Interventions including education and feed-back,^21^ electronic health records alerts^22^ and pharmacist-led HF medication titration clinics.^23^ These studies have proved the effectiveness of diverse interventions, including multi-disciplinary care and targeted physician education, in improving medication adherence and optimizing therapy for HF patients in primary care settings. There should be no doubt that focused interventions lead to an improvement of the GP’s adherence to GDMT in primary care settings. These previous studies have demonstrated a clear effect of interventions in controlled studies, using complex interventions in patients with HF mainly discharged from specialized care. Our results showed that a single educational visit, including a treatment conference between a cardiologist and the responsible GP resulted in an increase in GDMT and a decrease in healthcare contacts for patients with chronic stable HF (predominantly HFpEF). The results suggest that the dialogue might have increased the GPs’ knowledge in the management of HF patients, bridging the treatment gap between primary care and secondary care, and creating a path for future co-operation and discussion between disciplines.

## Conclusion

This intervention study in patients with chronic stable HF in primary care shows the potential of a cross-disciplinary approach in bridging the gap between cardiology and primary care. The implementation of the dialogue between primary care and cardiology was associated with improved GDMT adherence, especially for SGLT2i and ARNi, and a decreased number of ambulatory health care contacts.

## Supplementary Material

xvag036_Supplementary_Data

## Declarations

### Data availability

No data were generated or analysed for this manuscript.

### Pre-registered clinical trial number

NCT04129658
