# Supplementary material for: Bridging the gap in heart failure management: the effect of a cross-disciplinary intervention on guidelines-directed medical therapy in primary care
Source: ESC Heart Fail. 2026 Jan 20;13(1):xvag036. doi: 10.1093/eschf/xvag036 (PMC13108280; doi:10.1093/eschf/xvag036)
Supplement: xvag036_Supplementary_Data [file xvag036_supplementary_data.docx]

## Supplementary

Supplementary Table 1. Comparison of GDMT at recruitment and after treatment conference, stratified by patients with HFrEF and HFmrEF.

|  |  | GDMT after treatment conference | | p-value^a^ |
| --- | --- | --- | --- | --- |
| **HFrEF** |  | No | Yes | <0.001 |
| GDMT at | No | 91 | 31 |  |
| recruitment | Yes | 5 | 27 |  |
| **HFmrEF** |  | No | Yes | 0.020 |
| GDMT at | No | 136 | 12 |  |
| recruitment | Yes | 3 | 18 |  |

^a^McNemar’s test
